# Supplementary material for: Topological electromagnetic waves in dispersive and lossy plasma crystals
Source: Sci Rep. 2023 Nov 22;13:20445. doi: 10.1038/s41598-023-47848-5 (PMC10665461; doi:10.1038/s41598-023-47848-5)
Supplement: Supplementary file 1 — Supplementary Information. [file 41598_2023_47848_MOESM1_ESM.pdf]

# Topological electromagnetic waves in dispersive and lossy plasma crystals

Chen Qian, Yue Jiang, Jicheng Jin, Thomas Christensen, Marin Soljačić, Alexander V. Kildishev, Bo Zhen

## Material fitting in the ANSYS Lumerical FDTD

We use a finite-difference-time-domain (FDTD) approximation to the Maxwell equations, employing a commercial FDTD solver (ANSYS Lumerical FDTD). The Lumerical FDTD takes in the analytical (or experimental) data of complex permittivity and fits the data with a set of basis functions, consisting of the Drude-Lorentz model or a proprietary multi-coefficient model (MCM) [1]. In Fig. 1, the input data from the analytical description and a fitted curve from the Lumerical FDTD are presented. Along the $z$-direction, the data is retrieved from the analytical Drude model, so the fitted curve is indiscernible from the input data. The absorption part, i.e., $\Im(\varepsilon^{\pm})$ depicted in Fig. S1(d)-(e), is also fitted well. In contrast with $\Im(\varepsilon^{\pm})$, the real part of the fitted data $\Re(\varepsilon^{\pm})$ shown in Fig. S1(a)-(b) with solid blue lines deviates from the input data away from the resonance and at $f \to 0$. Around the resonant frequency, $\Re(\varepsilon^{+})$ matches the input data well. The insufficient accuracy of the internal approximation of the off-resonance plasma dielectric function does not allow for a detailed analysis of the plasma crystal bands.

**Simulation with** MEEP

MEEP [2], similar to ANSYS LUMERICAL FDTD, uses the FDTD method for most general computational electromagnetics and photonics problems. In contrast with the commercial ANSYS LUMERICAL FDTD, MEEP is an open-source software package. MEEP does not have a problem with the approximation of the plasma dielectric function, and we compute the band structures for the configuration shown in Fig. S3(a) with a given spatial resolution, defined as a ratio of lattice constant $a$ to the square grid size $\Delta a$, res $= \frac{a}{\Delta a}$.

Figure S2(a)-(d) compares the flat bands obtained with MEEP to the flat bands computed with a customized IRAM-based solver (COMSOL MUTIPHYCS™) and shown in Fig. 3(a). First, MEEP simulations confirm the presence of flat bands due to the material resonances below the resonance frequency. The FDTD method requires placing excitation sources into the structure to excite all possible modes. The mode profiles of these flat bands indicate that the corresponding modes are local resonances at the excitation points inside the plasma cylinder. Second, the flat bands do not converge with increasing resolution. In Fig. S3(a), the number of flat bands increases with increasing the number of mesh elements, and the flat bands are confined to certain regions. Figure S2(a)-(d) indicates that the flat bands obtained from MEEP are not well-confined with increasing the grid resolution, and the failure of convergence of these flat bands blocks the way to study the gaps and topologies of the dispersive bands.

## Extended angular momentum range of local resonances

In Fig. S3, the local resonances are plotted against their angular momenta. The figure extends the results depicted in Fig. 3(b) of the main text, by showing the modes with higher angular momenta.

For each band, the local resonances are plotted with dots similar to Fig. S3(b). As expected, the higher angular momentum modes are nonphysical due to mesh limitation. For example, for the flat bands in the upper region, the eigenfrequency should increase as the angular momentum magnitude increases, while the orange dots show a decreasing trend; for the lower region flat bands, they should be confined in the faded blue bands, while at large $m$, the modes move farther away from the faded blue bands.

## Edge states for $d/a$ = 0.74996

Figure S4 depicts edge states calculated for $d/a = 0.74996$. The solid red lines indicate the evolving edge states at the top, while the solid blue line represents the unchanged edge state at the bottom. As $d/a$ increases, both trivial edge states keep going down in frequency, traveling through the first bulk continuum and eventually disappearing at zero frequency.

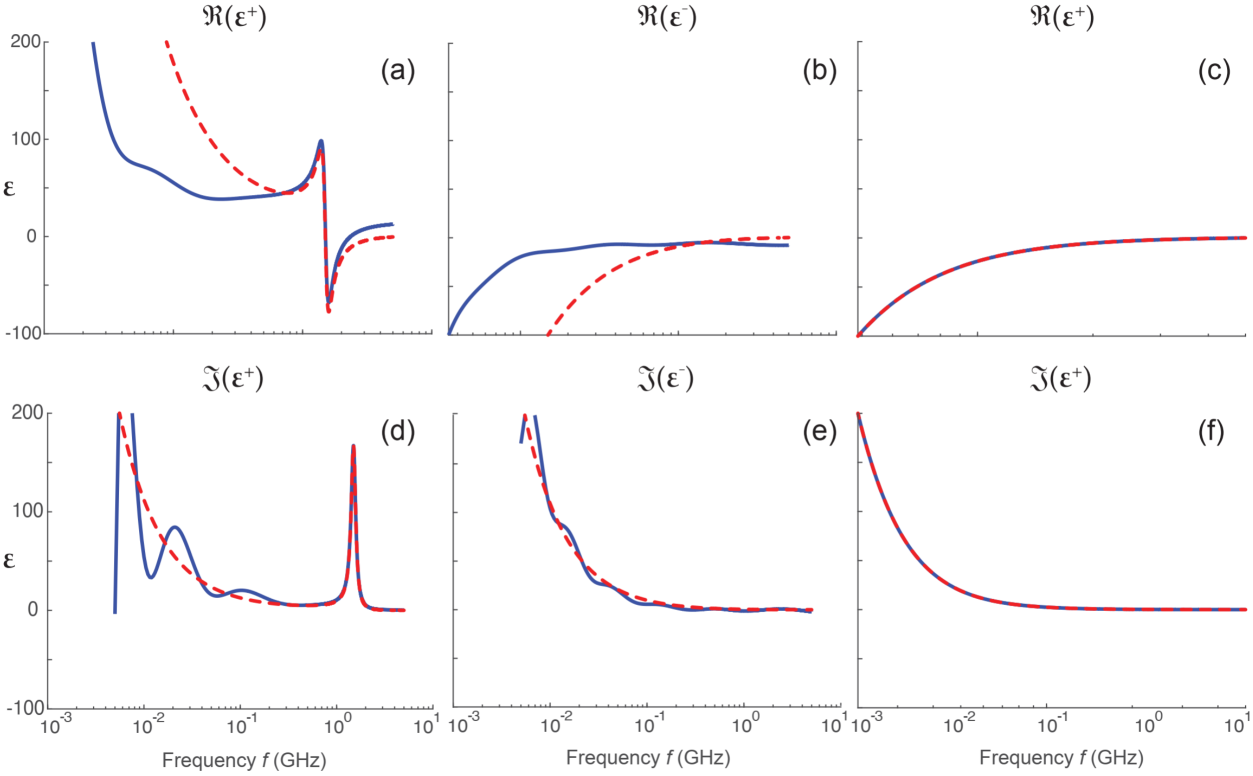


**Supplementary Figure S1 | The real and imaginary parts of the plasma permittivity components** ($\varepsilon^{+}, \varepsilon^{-}$, and$\varepsilon^{z}$) **and the matching curves, fitted with** ANSYS LUMERICAL FDTD**.** The dashed red lines in (a)-(f) represent the analytical data (see Eq. (8) in the main text), and the solid blue lines represent the fitted curves. (a)-(b) $\Re(\varepsilon^{\pm})$, (c) $\Re(\varepsilon^{z})$, (d)-(e) $\Im(\varepsilon^{\pm})$, and (f) $\Im(\varepsilon^{z})$.

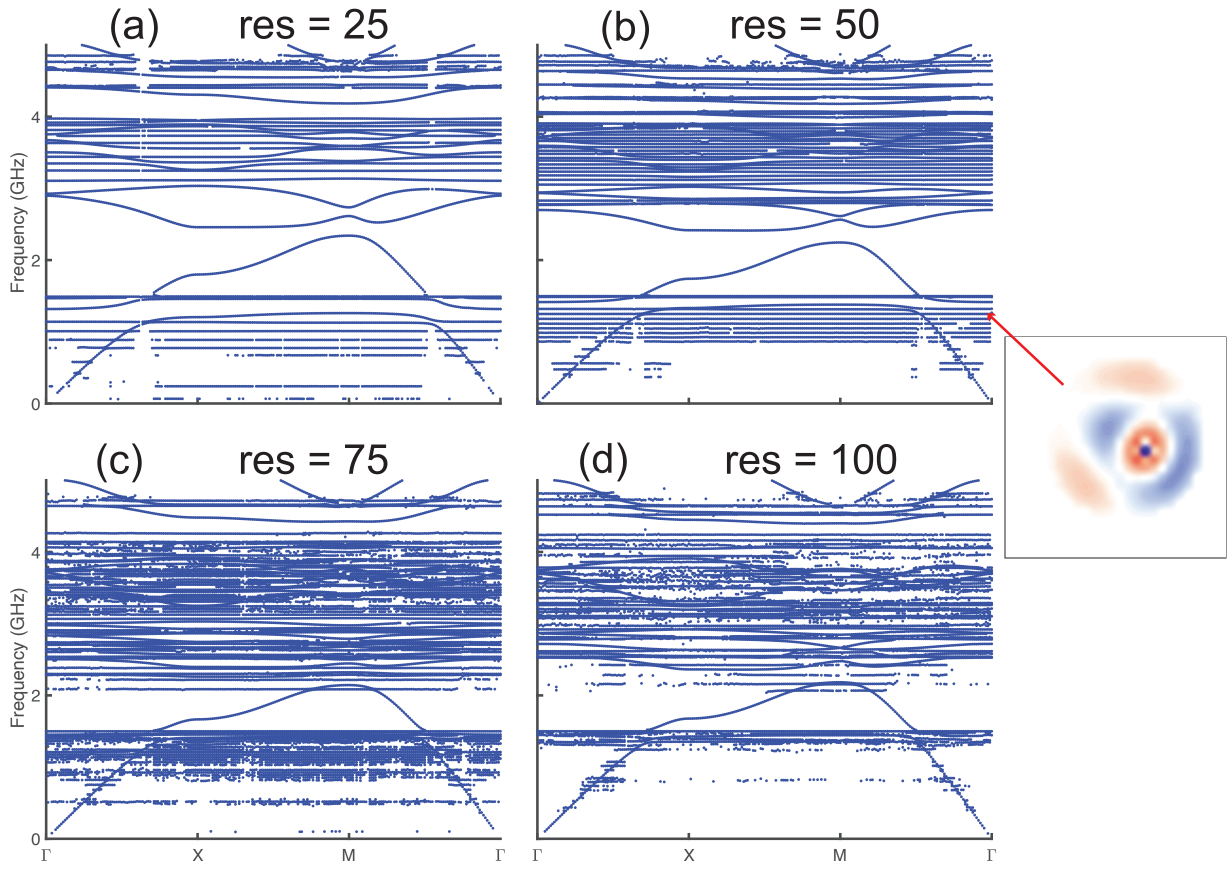


**Supplementary Figure S2 | The band structures with different grid resolutions.** The calculation is performed with the same configuration as Fig. 3(a), with lattice constant $a = 6\,\text{cm}$, and radius $r = 1.5\,\text{cm}$. We assume an external magnetic field of $B = 0.054\,\text{T}$ along the $z$-direction. (a)-(d) Calculated band structures with increasing spacial resolution, the mode profile of a local resonance at the source is shown in the inset.

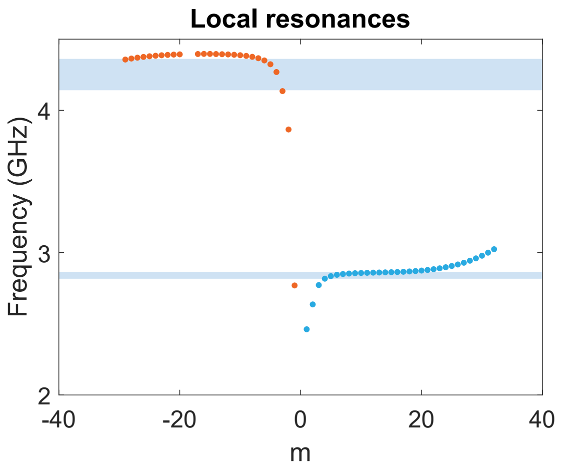


**Supplementary Figure S3 |** Supplement to the main text, Fig. 3(b). The orange dots represent the local resonances polarized counter-clockwise. The blue dots represent the local resonances polarized clockwise. The flat band regions are shown in faded blue.

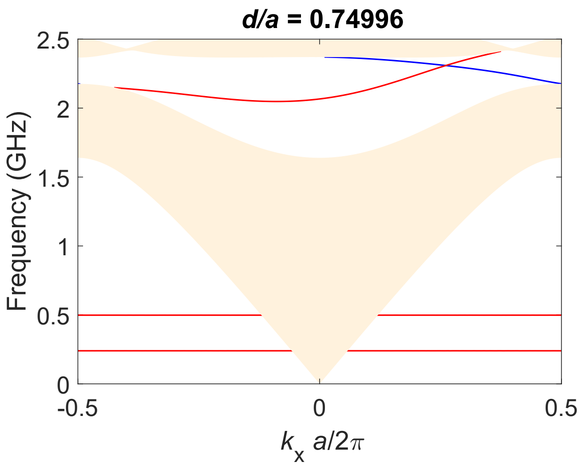


**Supplementary Figure S4 |** Supplement to the main text, Fig. 4. The red lines show the evolving edge states on the top side. The blue line represents the unchanged edge state at the bottom.

## References


1. ANSYS/LUMERICAL. White Paper: Overcoming the Multi-wavelength FDTD Challenge. https://www.lumerical.com/learn/whitepapers/ (accessed: Feb 15, 2023).

2. Oskooi, A. F. *et al.* Meep: A flexible free-software package for electromagnetic simulations by the fdtd method. *Computer Physics Communications* **181**, 687–702 (2010). URL https://www.sciencedirect.com/science/article/pii/S001046550900383X.
